# Supplementary material for: Ventromedial hypothalamus (VMHvl) nNOS neurons regulate social behaviors in a sex-specific manner
Source: Commun Biol. 2025 Dec 1;8:1732. doi: 10.1038/s42003-025-09279-y (PMC12672838; doi:10.1038/s42003-025-09279-y)
Supplement: Supplementary file 1 — Supplementary Information [file 42003_2025_9279_MOESM1_ESM.pdf]

Supplementary information

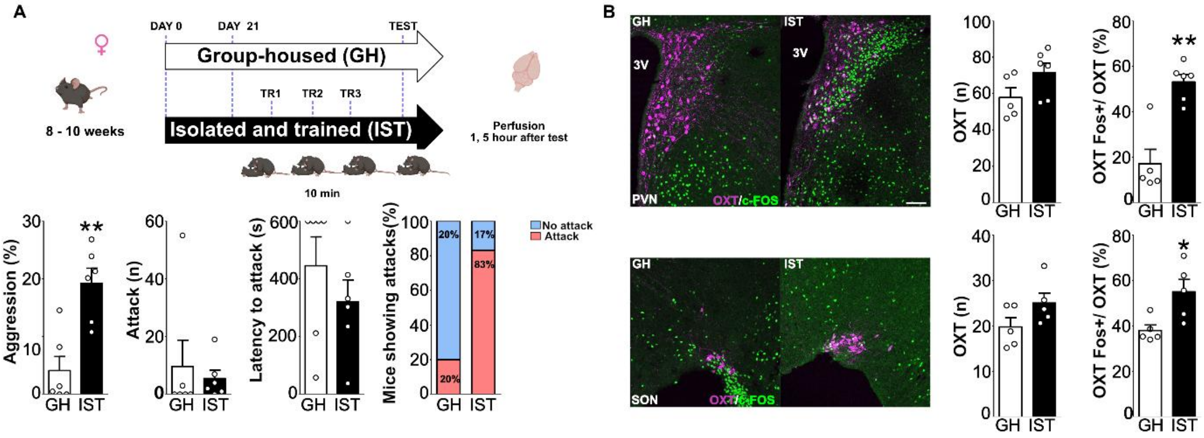

Supplementary figure 1. Female C57BL6/J mice were kept single-housed for 3 weeks before being confronted with a juvenile intruder (p16-21) for 3 consecutive days (A). Isolated and trained (IST, black bars n=6) mice displayed a higher percentage of time on aggression (two-tailed Student's t-test $t_{(10)}$= 4.24, p=0.01), but surprisingly did not show changes in number of attacks (Mann-Whitney U test U= 11.0, p=0.28) or attack latency (U= 12.0, p=0.37), but increased time spent on attacking (U= 4.0, p=0.02) when compared to GH controls (white bars n= 5-6). Furthermore, a higher proportion of IST females (83%) attacked the intruder compared to 20% of the GH mice (Fisher exact test, p<0.0001) (A). Additionally, as hypothesized by us, IST females (exhibited a higher percentage of oxytocin-positive neurons (magenta) co-expressing cFOS (green) in the paraventricular (PVN, U= 1.0, p=0.008) and supraoptic nucleus of the hypothalamus (SON, $t_{(18)}$= 2.82, p=0.02) (B). *p<.05; **p<.01 vs GH. Data are presented as mean + s.e.m. Scale bar 100µm.

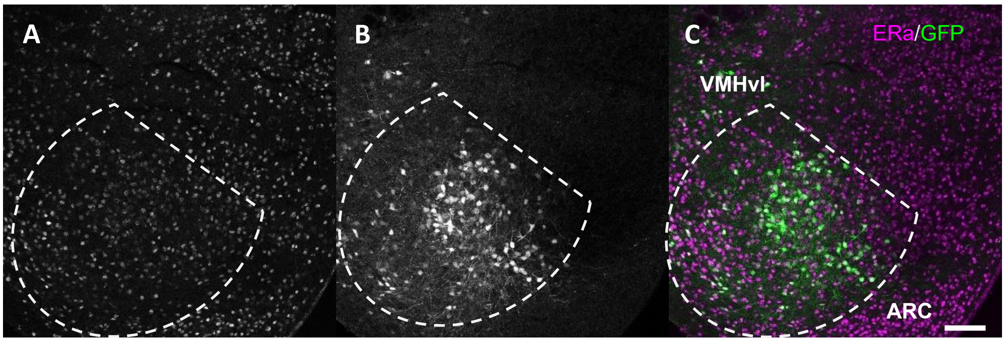

Supplementary figure 2: Image depicting viral infection, GFP expression (green) coming from control virus infusions (B) is constrained to the VMHvl with no leakage into the arcuate nucleus (ARC) below (Er**α** , **A,** in magenta). Scale bar 100µm.

Supplementary Table 1: Overview of detailed statistical analysis as well as p-values for data displayed in Figures 1 and 2. Significant p-values are in bold. For histological analysis, data are expressed as mean ± SEM.

Figure	Data	Detailed Statistics	pvalue	Numbers
**1b**	ERα	Mann-Whitney U test U= 0.0	**0.015**	Female= 4<br>Male= 5
	nNOS	two-tailed Student’s t-test $t_{(7)}$=0.69	0.512	
	Erα&nNOS/ nNOS (%)	two-tailed Student’s t-test $t_{(7)}$=3.44	**0.01**	
**1c**	ERα	two-tailed Student’s t-test $t_{(6)}$=15.81	**<0.0001**	
	nNOS	two-tailed Student’s t-test $t_{(6)}$=22.02	**<0.0001**	
	Erα&nNOS/ Erα (%)	two-tailed Student’s t-test $t_{(6)}$= 8.24	**0.0002**	
	Erα&nNOS/ nNOS (%)	Mann-Whitney U test U= 0.0	**0.028**	
**2d**	Number of nNOS neurons	Object= 112.8±18.9 Behavior: 56.± 10.8<br>Mann-Whitney U test U= 2.0	*0.06*	
	c-Fos&nNOS/nNOS (%)	Mann-Whitney U test U= 0.0	**0.031**	Object=4<br>Behavior=5
	c-Fos&nNOS/nNOS (n)	Object= 9.0±0.5 Sex: 8.2 ± 0.5<br>Mann-Whitney U test U= 5.5	0.31	
	pERK&nNOS/nNOS (%)	Mann-Whitney U test U= 0.0	**0.015**	
	pERK&nNOS/nNOS (n)	Object= 3.1±1.2 AGG: 6.8 ± 0.7<br>Mann-Whitney U test U=2	*0.06*	
	c-FOS&pERK&nNOS/nNOS (%)	Mann-Whitney U test U= 2.0	0.063	
	pERK/c-FOS (%)	Mann-Whitney U test U= 4.0	0.19	
**2f**	c-Fos&nNOS/nNOS (%)	Mann-Whitney U test U= 0.0	**0.009**	Object=6<br>virg/Behavior=4<br>Lac=3<br>IST=8
	c-Fos&nNOS/nNOS (n)	Object= 7.1±0.8 Sex: 12.9 ± 2.7<br>Mann-Whitney U test U= 5.5	**0.0381**	
	pERK&nNOS/nNOS (%)	Mann-Whitney U test U= 3.0	0.238	
	c-FOS&pERK&nNOS/nNOS (%)	Mann-Whitney U test U= 8.0	0.438	
	Reproductive and aggressive states	Kruskal-Wallis test, K=6.66	0.157	
	Reproductive and aggressive states pERK&nNOS (n)	Object= 5.1±1.0 virgin=2.6±1.3 Lac=7.1±3.1 and IST=6.6±3.0<br>Kruskal-Wallis test, K= 2.92	0.404	
	Reproductive states nNOS number nNOS (n)	Object= 101.4±10.5 virgin= 93.3±3.7 Lac= 63±0.2 and IST= 104.7±9.7<br>Kruskal-Wallis test, K= 5.94	0.115	

Supplementary Table 2: Overview of detailed statistical analysis as well as p values for behavioral data displayed in Figure 3. Significant p values are in bold and nearly significant in italics.

Figure	Data	Detailed Statistics	pvalue	Numbers
**3b**	nNOS (n)	Mann-Whitney U test U= 0.00	**0.002**	Ctrl= 5 nNOS-del=7
	nNOS (n)/Aggression	Pearsons correlation r= 0.636	**0.001**	
	Mice attacking (%)	Fisher's exact test	**0.0001**	Ctrl= 7 nNOS-del=9
	2 way ANOVA followed by Bonferroni's multiple comparisons test			
	Aggression (%)	Virus effect: $F_{(1, 13)} = 28.55$	**0.0001**	
		Training effect: $F_{(2, 26)} = 3.621$	**0.04**	
		Virus x training effect: $F_{(2, 26)} = 0.04564$	0.95	
	Attack number	Virus effect: $F_{(1,13)}= 37.11$	**<0.0001**	
		Training effect: $F_{(2,26)}= 0.9976$	0.38	
		Virus x training effect: $F_{(2,26)}=0.5755$	0.57	
	Attack latency	Virus effect: $F_{(1,13)}=19.58$	**0.0007**	
		Training effect: $F_{(2,26)}=0.2474$	0.78	
		Virus x training effect: $F_{(2,26)}= 0.408$	0.86	
**3d**	Mounts& Intromissions	Virus effect: $F_{(1,13)}=9.242$	**0.009**	
		Training effect: $F_{(2,26)}= 5.690$	**0.008**	
		Virus x training effect: $F_{(2,26)}=2.29$	0.12	
	Intromission Latency	Virus effect: $F_{(1,14)}=5.584$	**0.03**	
		Training effect: $F_{(2,28)}=3.031$	0.06	
		Virus x training effect: $F_{(2,28)}=0.5977$	0.55	
**3c**	Social preference	One sample t-test ctrl: t-test $t_{(6)}=2.49$	**0.047**	
		One sample t-test nNOS-del: t-test $t_{(5)}=3.35$	**0.01**	
	Mate preference	One sample t-test ctrl: t-test $t_{(5)}=2.49$	**0.04**	
		One sample t-test nNOS-del: t-test $t_{(8)}=0.26$	0.79	
**3e**	Mounts	two-tailed Student's t-test $t_{(13)}=2.147$	0.05	
	Intromissions	two-tailed Student's t-test $t_{(13)}=2.759$	**0.01**	
	Copulation Latency	Mann-Whitney U test U= 10.0	**0.049**	
	Mounts + Intromissions/ nNOS (n)	Pearsons correlation r= 0.3634	**0.038**	

Supplementary Table 3: Overview of detailed statistical analysis as well as p values for behavioral data displayed in Figure 4. Significant p values are in bold and nearly significant in italics.

Figure	Data	Detailed Statistics	pvalue	Numbers
**4f**	nNOS (n) intact	Mann-Whitney U test U= 0.00	**0.0006**	Ctrl= 7 nNOS-del=7
	nNOS (n) ovx+E2	Mann-Whitney U test U= 0.00	**0.0043**	Ctrl=5 nNOS-del=7
2 way ANOVA followed by Bonferroni's multiple comparisons test				
**4b**	Aggression (%)	Virus effect: $F_{(1, 14)} = 0.2751$	0.61	Ctrl= 8 nNOS-del=8
		Training effect: $F_{(2, 28)} = 0.9523$	0.39	
		Virus x training effect: $F_{(2, 28)} = 0.9207$	0.41	
	Attack number	Virus effect: $F_{(1,14)} = 0.008434$	0.92	
		Training effect: $F_{(2,28)} = 1.897$	0.17	
		Virus x training effect: $F_{(2,28)} = 0.3862$	0.68	
	Attack latency	Virus effect: $F_{(1,14)} = 0.05085$	0.82	
		Training effect: $F_{(2,28)} = 2.671$	0.086	
		Virus x training effect: $F_{(2,28)} = 0.3224$	0.72	
**4d**	Social investiga-tion (%)	Virus effect: $F_{(1,14)} = 1.298$	0.27	
		Training effect: $F_{(2,28)} = 0.08765$	0.91	
		Virus x training effect: $F_{(2,28)} = 0.5256$	0.59	
**4e**	Lordosis (%)	Virus effect: $F_{(1,12)} = 34.29$	**<0.0001**	Ctrl= 7 nNOS-del=7
		Training effect: $F_{(2,24)} = 1.305$	0.29	
		Virus x training effect: $F_{(2,24)} = 4.969$	**0.01**	
	Lordosis (%) / nNOS (n)	Pearsons correlation r= 0.872	**<0.0001**	
**4c**	Social preference	One sample t-test ctrl: t-test $t_{(11)} = 2.345$	**0.038**	Ctrl= 12 nNOS-del=14
		One sample t-test nNOS-del: t-test $t_{(13)} = 1.87$	0.08	
	Mate preference	One sample t-test ctrl: t-test $t_{(12)} = 1.0$	0.33	
		One sample t-test nNOS-del: t-test $t_{(13)} = 4.44$	**0.0007**	
		One sample t-test ctrl P-E: t-test $t_{(5)} = 2.63$	**0.046**	
		One sample t-test nNOS-del P-E: t-test $t_{(3)} = 0.2093$	0.12	
		One sample t-test ctrl-MD: t-test $t_{(7)} = 0.28$	0.78	
		One sample t-test nNOS-del-MD: t-test $t_{(9)} = 3.971$	**0.003**	

Supplementary Table 4: Detailed statistical analysis as well as p values for data related to Experiment D. Significant p values are in bold and nearly significant in italics.

Figure	Data	Mean±SEM	pvalue	Numbers
**Males Wilcoxon Test**				
**Aggression (%)**	Pre-test	14.45±3.86	W= -7.0 p=0.43	N=6
	Vehicle	8.88±4.25		
	SNAP	3.89±2.77		
**Attacks (n)**	Pre-test	7.33±2.27	W= -10.0 p=0.12	
	Vehicle	4.5±2.39		
	SNAP	2.0±1.48		
**Attack latency (s)**	Pre-test	320±72.85	W= 9.0 p=0.31	
	Vehicle	458.3±64.83		
	SNAP	478.8±30.29		
**Mounts + Intromissions (n)**	Pre-test	23.83±8.52	W= -18.0 p=0.09	
	Vehicle	35.71±22.98		
	SNAP	8.67±6.47		
**Females Wilcoxon Test**				
**Lordosis (%)**	Pre-test	7.14±4.26	W= 9 p=0.37	N=7
	Vehicle	7.14±4.26		
	SNAP	15.71±8.95		
Males	nNOS (n)	2±1		
Females	nNOS (n)	15.13±7.025		
