# Supplementary material for: Ventromedial hypothalamus (VMHvl) nNOS neurons regulate social behaviors in a sex-specific manner
Source: Commun Biol. 2025 Dec 1;8:1732. doi: 10.1038/s42003-025-09279-y (PMC12672838; doi:10.1038/s42003-025-09279-y)
Supplement: Supplementary file 4 — Description of Additional Supplementary files [file 42003_2025_9279_MOESM4_ESM.pdf]

Description of Additional Supplementary files

File name: Supplementary data 1

Description: Supplementary Dataset with single values used to build panels in all figures included in the manuscript

File name: Supplementary Video 1

Description: Video depicting female aggressive behavior
